# Supplementary material for: A set of multi-entry identification keys to African frugivorous flies (Diptera, Tephritidae)
Source: Zookeys. 2014 Jul 24;(428):97–108. doi: 10.3897/zookeys.428.7366 (PMC4143993; doi:10.3897/zookeys.428.7366)
Supplement: Supplementary material 6 — Key to Ceratitis [file zookeys-428-097-s006.zip › SF6_ZooKeys_key to Ceratitis/key/SF6_ZooKeys_key to Ceratitis/Media/Html/Ceratitis ditissima.htm]

Ceratitis (Pardalaspis) ditissima (Munro) 


***Ceratitis*** **(*Pardalaspis*) *ditissima*
(Munro)**

*Pardalaspis* *ditissima*
Munro, 1938: 164.

Body
length: 6.79 (6.0-7.5) mm; wing length: 6.79 (6.0-7.5) mm.

Male

Head.
Antennal segments orange to dark red. Third antennal segment twice as long as
second segment. Arista basal part orange, otherwise dark. Frons yellow in
ground colour (in some specimens orange-red), silvery over entire length; with
dispersed short hairs, same colour as frons. Ocellar triangle dark. Face dark
red; no median band but lower margin white. Occiput moderately swollen below,
pale whitish. Chaetotaxy normal for subgenus.

Thorax.
Ground colour of mesonotum greyish-brown, anterior border dark brown,
sickle-shaped and extended along lateral margins; also with three poorly
defined darker stripes. Postpronotum paler than mesonotum. Chaetotaxy normal
for subgenus. Mesonotum with pale pilosity, brown part with dark hairs.
Anepisternum along upper margin and lower half with darker hairs. Two
anepisternal bristles, sometimes three, but then median one usually less well
developed.� Scutellum yellow with apical markings black; basally with two dark
spots.

Legs
dark yellow to orange brown, femora with darker streaks; setation typical of
subgenus. Hind femur with dorsal hairs dark apically. Setae general dark in
colour, posterior row on front femur dark.

Wings
with brownish bands, yellow markings strongly reduced. Banding, setation and
venation normal for subgenus. Marginal band continuous; discal band joined with
marginal band; cross-vein r-m at middle of discal cell; vein R1 ending before
cross-vein r-m.

Abdomen.
grey-brown, sometimes with orange tinge, with clearly defined spots. Pattern of
spots and setation normal for subgenus.

Female

As
male except for the following characters. Frons yellow, not silvery; with
distinctly darker hairs. Face wholly yellow, lower margin same colour.
Mesonotum without darker anterior part, concolorous; pilosity completely pale.
Scutellum basally sometimes more whitish.� Oviscape shorter than abdominal
terga 3-6, orange in colour.

(Description
after De Meyer, 1996)
